# Cecal epidermoid cyst: a neonatal case with clinicopathological consideration

**DOI:** 10.1186/s12887-021-02884-w

**Published:** 2021-09-20

**Authors:** Joo-Young Na, Soo-Hong Kim, Narae Lee

**Affiliations:** 1 Department of Pathology, Pusan National University Yangsan Hospital, Yangsan, Republic of Korea; 2 Department of Pediatric Surgery, Pusan National University Children’s Hospital, Yangsan, Republic of Korea; 3 Department of Pediatrics, Pusan National University Children’s Hospital, 20, Geumo-ro, Mulgeum-eup, 50612 Yangsan, Republic of Korea

**Keywords:** Congenital, Cecum, Epidermoid cyst, Neonate

## Abstract

**Background:**

Only 10 cases of cecal epidermoid cyst (CEC) have been reported in the literature. Furthermore, its pathogenesis remains unclear. We report a rare case of congenital CEC in neonate, and discuss its clinicopathological findings.

**Case presentation:**

A cystic lesion was incidentally identified in the retroperitoneal area of the abdominal right lower quadrant during a routine prenatal ultrasonography (US), prompting an ileocolectomy 3 days after birth. This congenital cyst was composed of mucosal lining cells and submucosal connective tissues, and the inner lining mucosa was composed of stratified squamous epithelium and focally mucin-producing ciliated epithelium. Based on the macroscopic and microscopic findings, the cystic lesion was diagnosed as a congenital cecal epidermoid cyst.

**Conclusions:**

The management of a fetal abdominal mass should be tailored individually, considering that epidermoid cysts can occur in the cecum during the perinatal period. We report the clinicopathological findings in this case, including its possible pathogenesis.

## Background

In the literature, only 10 case of cecal epidermoid cysts (CECs) have been reported [1, 2], with its pathogenesis remaining unclear. CEC can be congenital or acquired in origin. In the congenital form, it is thought to develop from ectodermal implantation during embryogenesis and development, whereas in the acquired form, it is thought to develop from epithelial implantation secondary to previous trauma or surgery [3, 4]. Additionally, epidermoid cysts are more commonly found in the mediastinum, head and neck, sacrococcygeal area, central nervous system, and gonads [3]. Thus, we report a case of congenital CEC and discuss its clinicopathological findings and possible pathogenesis.

## Case presentation

A 28-year-old woman underwent regular follow-ups at an antenatal clinic, without needing any medication. At 24 weeks of gestation, an intra-abdominal cystic mass, approximately 2.0 × 1.5 cm in size, was found during routine prenatal ultrasonography (US). Its size increased to 3.5 × 2.4 cm at 32 weeks of gestation, and thereafter, it ceased to grow until delivery. The amniotic fluid index at 32 weeks of gestation was 1.5 cm, and this was maintained at 1.3 cm by 36 weeks of gestation. Eventually, the male neonate was born at 38 weeks and 2 days of gestation via normal spontaneous vaginal delivery, with Apgar scores of 9 and 10 at 1 and 5 min, respectively. He had a body weight of 3500 g (50^th^ percentile), height of 49.0 cm (45th percentile), and head circumference of 35.5 cm (55th percentile). Initial physical examination revealed a soft abdomen with no distention.

**Fig. 1:**
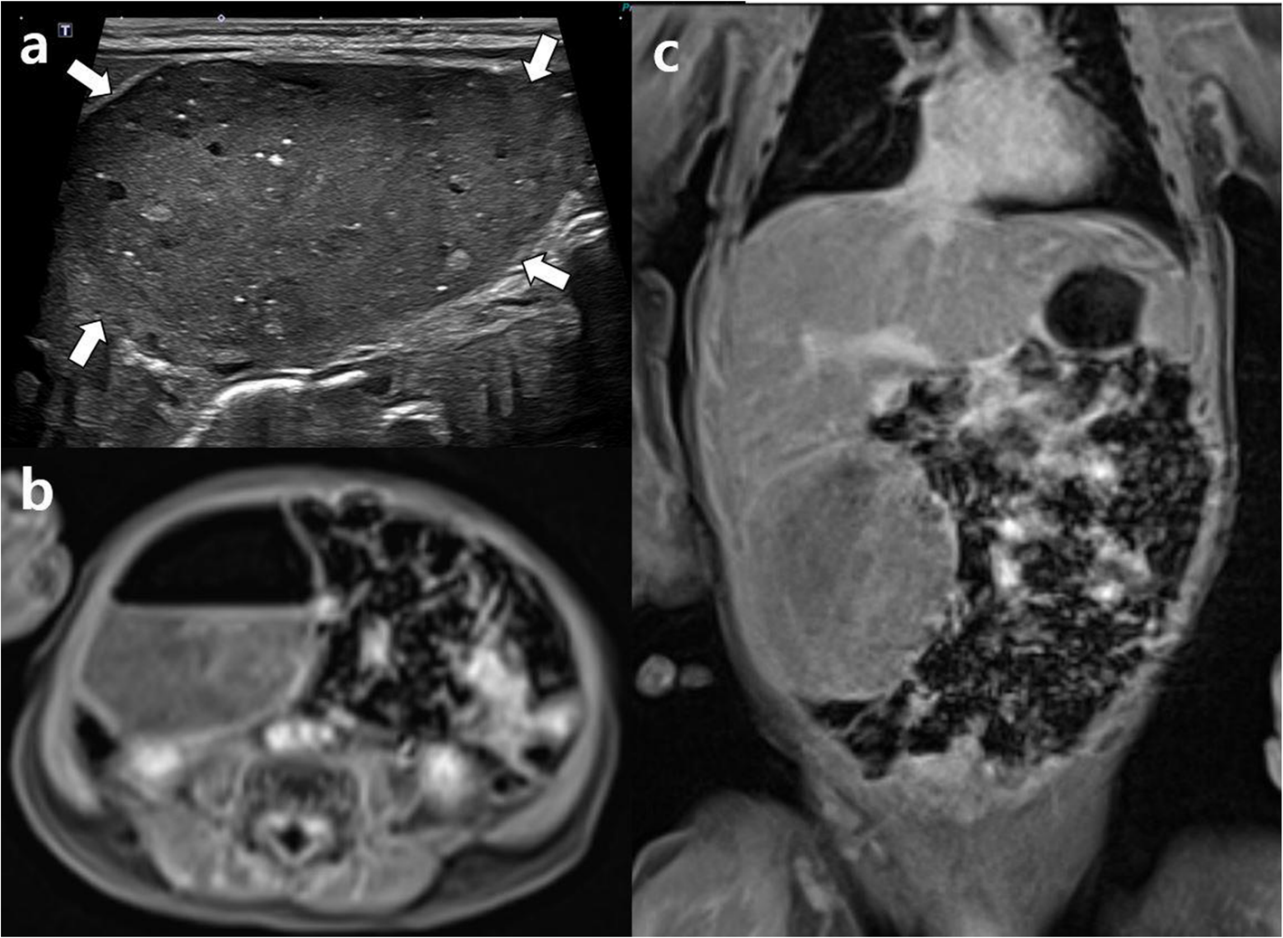
**a** A transverse sonogram shows a cyst with echogenic material, fluid, and calcification (arrows). It originated from the retroperitoneum and is located in front of the inferior vena cava and descending aorta. The size of the cyst is 4.2 × 2.3 × 4.2 cm. Contrast-enhanced fat-saturated T1-weighted MR image of transverse (**b**) and coronal (**c**) view reveal a cystic mass with air-fluid level and multiple tiny low signal intensity component. Size of cystic mass is 4.6 × 4.5 × 5.3 cm

**Fig. 2:**
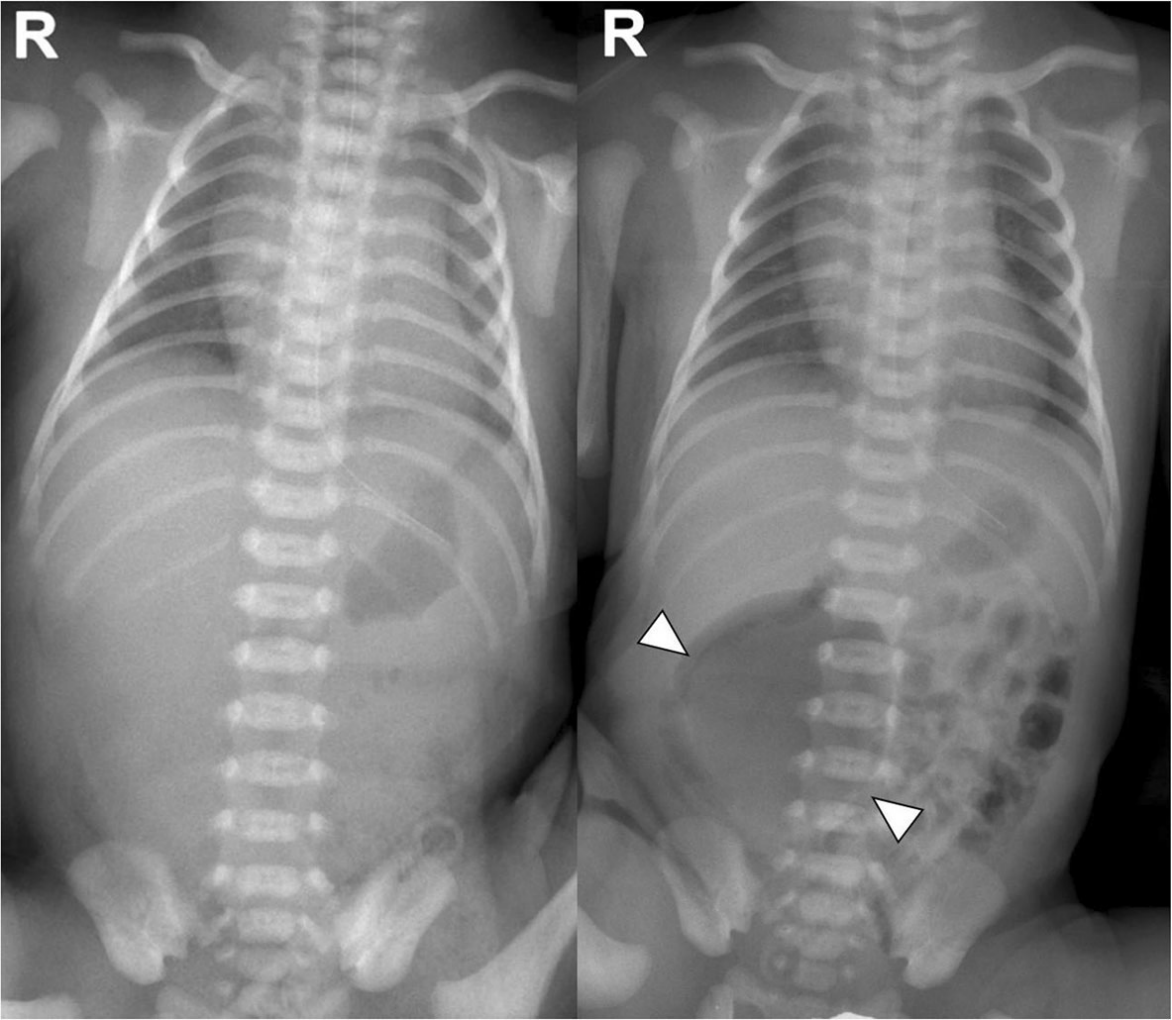
Infantography shows a nonspecific bowel gas pattern 1 h after birth (left). Eight hours after birth, a radiolucent lesion at the abdominal right lower quadrant appears, with an interval increase in the size of the radiolucent lesion to 5.2 × 5.1 cm at 48 h after birth (arrowheads) (right)

**Fig. 3:**
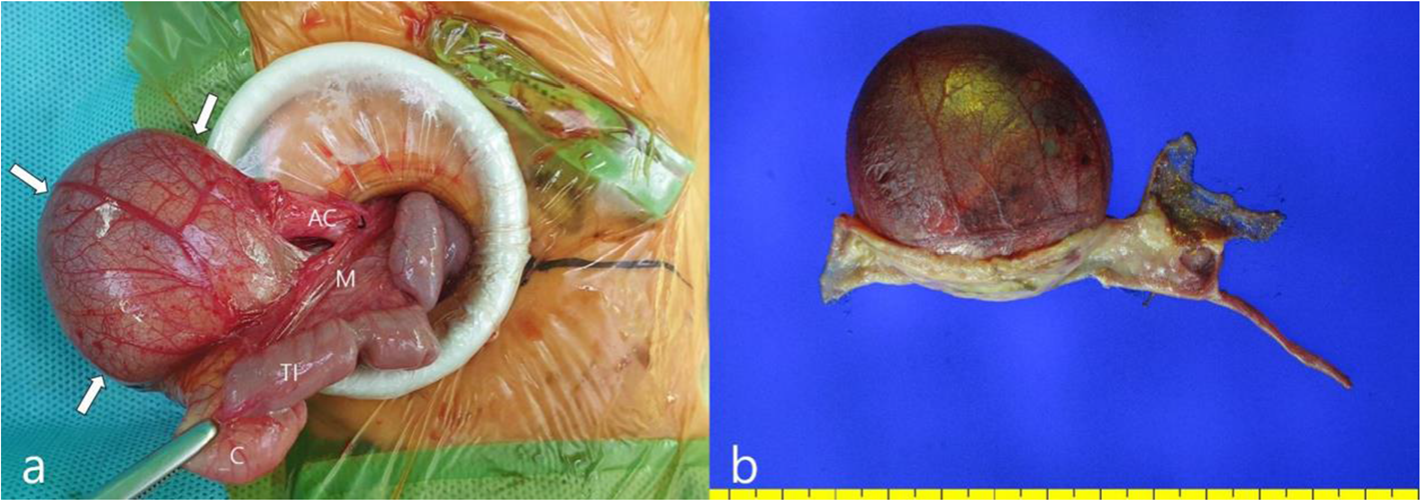
**a** Intraoperative photograph of the cyst (arrows), and a macroscopic examination shows (**b**) a cyst measuring 5.0 × 6.6 cm in size is noted, which is attached at the serosal side of the cecum. There is no connection between the cyst and the cecal lumen on macroscopic examination. *AC: Ascending colon; C: Cecum; TI: Terminal Ileum; M: Mesentery*

Abdominal US performed 25 h after birth showed a large cyst (4.2 × 2.3 × 4.2 cm) with echogenic material, fluid, and calcification, which originated from the retroperitoneum and was located in front of the inferior vena cava and descending aorta (Fig. 1a). Due to the echogenic shadowing of the calcific components, we decided to perform magnetic resonance imaging (MRI) to rule out the possibility of a retroperitoneal teratoma. At 2 days of age, contrast-enhanced MRI of the abdomen was performed for further evaluation. Pre-contrast coronal T2-weighted MRI showed an intermediate to high signal intensity (SI) of a heterogeneous mass with a small low SI component. Further contrast-enhanced fat-saturated T1-weighted MRI of the coronal and transverse views revealed a cystic mass (4.6 × 4.5 × 5.3 cm) with an air-fluid level and multiple minute low SI components (Fig. 1b, c). Moreover, an increasing radiolucent cystic mass in the right lower quadrant (RLQ) began to appear 8 h after birth on serial infantography (Fig. 2). The patient underwent emergency laparotomy performed by a pediatric surgeon on day 3 of life. Under general anesthesia, the cystic mass was exposed, and over 20 mL of air and dark brown mucinous fluid were aspirated through a syringe (Fig. 3a). The cystic mass was attached to the cecum, prompting the need for an ileocolectomy with end-to-end anastomosis. Five days post-surgery, the patient started feeding with mother’s milk, and the postoperative course was uneventful. He was discharged 10 days after the surgery without any complications.

**Fig. 4:**
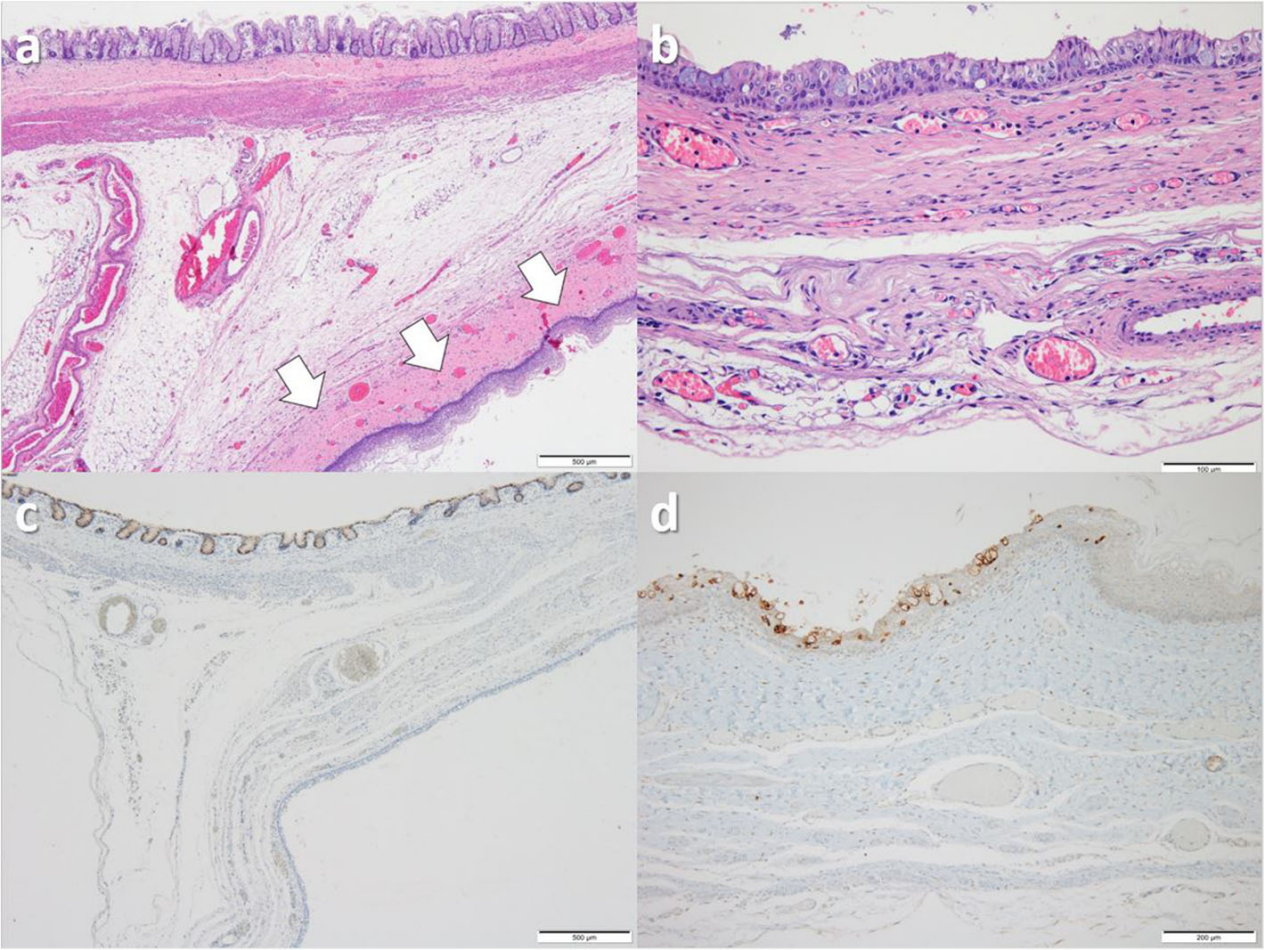
The cyst is located at the subserosal layer of the cecal wall (arrows), which is composed of stratified squamous epithelium and submucosal connective tissues (**a**, × 40), and the inner mucosal lining is focally composed of mucin-producing ciliated epithelium in some foci (**b**, × 200). Immunohistochemical staining shows that the cecal mucosa is reactive to MUC2 (**c**, × 40), while the cystic mucosa is reactive to MUC5AC (d, × 100)

On macroscopic examination, a cyst was identified without any macroscopic connection to the cecal lumen. The cyst contained a dark brown mucinous fluid, and the mucosal and serosal surfaces of the cecum were unremarkable (Fig. 3b). On microscopic examination, cyst wall was located at the subserosal layer of the cecal wall, composing of mucosal lining cells and submucosal connective tissues. The cyst cell lining was composed of mature, keratinized, or non-keratinized stratified squamous epithelium and focally mucin-producing ciliated stratified epithelium; however, there were no findings of gastrointestinal mucosal epithelium in the entire cyst and there was no smooth muscle outer layer in the cystic wall, except for an attached portion of the cecal wall. Transition areas between the squamous and mucin-producing ciliated epithelia were noted in some foci. Immunohistochemical examination revealed that the colonic mucosa was reactive to MUC2, while the mucin-producing ciliated epithelium of the cystic mucosa was reactive to MUC5AC (Fig. 4). The boy has had no further symptoms and is doing well. We are scheduled to follow up at age 6 months with abdominal US.

## Discussion and conclusions

The present report describes a case of CEC in a newborn. Only 10 CECs have been reported in English literature, with the only one pediatric patient being an 8-year-old girl [1, 2]. Epidermoid cysts are generally considered sequestration cysts that may be congenital, but some CEC cases were attributed to iatrogenic implantation of epidermal fragments by surgical devices during the previous abdominal operation, such as appendectomy or cesarean surgery [2]. With recent advances in US techniques, fetal abdominal cysts are being reported more often, with ovarian cysts as the most common type in the fetal period [5]. CEC clinical diagnosis remains difficult because it can be confused with ovarian cysts, lymphatic cysts, mesenteric cysts, intestinal duplication, appendiceal mucocele, and meconium pseudocyst, especially if the diagnosis is established in the prenatal period [6]. The treatment of choice for CEC is surgical resection. Since residual squamous epithelial tissues may lead to recurrence and have malignant potential, complete removal is warranted [7, 8]. Although accurate preoperative diagnosis allows appropriate surgical planning and provides a good prognosis, this becomes difficult for neonatal CECs.

Two pathogenic mechanisms have been proposed for congenital sequestration. One mechanism suggests that ectodermal implantation occurs at the time of neural groove closure during embryogenesis; however, some researchers have postulated that given the location of the cecum and the neural groove or other epithelial fusion lines, this mechanism would be unusual [3]. Another mechanism is that the origin of the congenital heterotopic tissue may have transpired during the intrauterine rotation of the gut back into the abdominal cavity, especially since the cecum is one of the last elements to re-enter the abdomen [4]. Furthermore, the fact that most CECs occur in the subserosal layer supports this mechanism. In the present case of congenital CEC, heterotopic epithelial tissue was located in the subserosal layer, which is suggestive of ectodermal implantation during intrauterine rotation of the gut. Additionally, mucin-producing ciliated stratified epithelium was identified in this case and this epithelium resembled a bronchial epithelium. This finding could suggest a possible pathomechanism which may be related to the bi-differentiation of heterotopic ectodermal inclusion tissue. We hypothesized that ectodermal tissue was sequestrated in the subserosal layer of the cecum during the rotation of the gut back into the abdominal cavity, wherein these tissues differentiated into both squamous and mucin-producing ciliated epithelium. A previous report suggested that the most likely explanation for CEC development and its sharing of the cecal muscular wall is the result of aberrant embryonic ectodermal implantation during embryogenesis [9]. Our present case showed a subserosal cyst composed of squamous mucosa and submucosal connective tissue, which supports the aforementioned proposed mechanism. Furthermore, immunohistochemical phenotypes of cecal and cystic mucosal epithelium were different; colonic mucosa was reactive to intestinal mucin MUC2, while the mucosa lining the cyst wall was reactive to gastric and bronchial mucin MUC5AC. When we evaluated the serial radiographs in this case, the RLQ intraluminal gas, which initially did not appear until after birth, continuously expanded. These findings suggest that the cyst was connected to the intestinal lumen, even though the macroscopic examination revealed no connection between two. On microscopic examination, there was one focus of the junction between the cecal and squamous epithelium which was located in the cecal muscularis propria, suggesting a microscopic fistula. Thus, we assumed that the two lumens may have been connected through the microfistula.

In conclusion, considering that epidermoid cysts can develop in the cecum during the perinatal period, the management of a fetal abdominal mass should be tailored individually. Moreover, the possibility of CEC should be considered in the differential diagnosis of subserosal cysts in the cecal area, and the cyst should be completely removed to achieve a good prognosis.

## Data Availability

All data generated or analyzed during this study are included in this published article.

## Abbreviations

CEC: Cecal epidermoid cyst
MRI: Magnetic resonance imaging
MUC2: Mucin 2
MUC5AC: Mucin 5AC
RLQ: Right lower quadrant
SI: Signal intensity
